# Characterization of a novel composite ICE in *Streptococcus agalactiae* conferring resistance to macrolides [*erm*(TR)] and cadmium (*cadA*)

**DOI:** 10.3389/fmicb.2026.1763839

**Published:** 2026-03-09

**Authors:** Sida Yi, Chunli Shi, Liufan Yin, Xing Xu, Xueliang Wang

**Affiliations:** 1 Department of Molecular Biology, Shanghai Center for Clinical Laboratory, Shanghai, China; 2 Institute of Antibiotics, Huashan Hospital, Fudan University, Shanghai, China; 3 Department of Molecular Diagnostic Innovation Technology, Shanghai Academy of Experimental Medicine, Shanghai, China

**Keywords:** antibiotic resistance genes, antimicrobial susceptibility, cadmium resistance, integrative and conjugative elements (ICEs), *Streptococcus agalactiae*

## Abstract

**Background:**

Macrolide resistance genes (*erm* and *mef* families) and heavy metal resistance genes (*cadA*) are increasingly disseminated among streptococci via diverse mobile genetic elements.

**Methods:**

Through whole-genome sequencing of 16 *Streptococcus agalactiae* isolates resistant to both erythromycin and clindamycin, we identified 19 integrative and conjugative elements (ICEs), a type of self-transfer genetic elements, conferring antibiotic resistance. Among these, a novel composite ICE designated ICE*Sag*39 was identified in *S. agalactiae Sag*39 through comparative analysis with the NCBI database.

**Results:**

ICE*Sag*39 measured 113,125 bp in length, and it featured a nested “Russian doll” structure comprising an ICE*Sa*2603 family backbone integrated with an internal Tn*1806*-like ICE. The embedded Tn*1806*-like ICE contained four variable regions (VR1-VR4) that serve as insertion hotspots; among these, VR3 and VR4 carry *erm*(TR) from ICE*Sp*2907 and the cadmium resistance gene *cadA* from an uncharacterized genetic element, respectively. Conjugation and excision assays confirmed that ICE*Sag*39 transfers at a frequency of 8.2 × 10^−9^ and co-transfers both resistance determinants. Under cadmium stress, transconjugants carrying ICE*Sag*39 displayed enhanced growth relative to the recipient. Although the internal Tn*1806*-like ICE was also capable of independent transfer, its efficiency was significantly lower (< 10^−9^), and its circular form is undetectable by PCR. Database screening identified 199 structurally similar ICEs (ICE*Sag*39-like ICE), 62.8% (125/199) of which co-carried *erm*(TR) and *cadA*, underscoring the prevalence of this ICE and its associated resistance traits.

**Conclusions:**

This study characterizes a new composite ICE and elucidates a modular mechanism that facilitates the assembly and dissemination of resistance genes, thereby promoting bacterial genome diversification.

## 1 Introduction

Macrolide resistance in streptococci is primarily mediated through two distinct mechanisms: active drug efflux and ribosomal target site modification (Leclercq, 2002; Varaldo et al., 2009). The first mechanism involves efflux pumps encoded by *mef* genes [such as *mef* (E), *mef* (A), and *mef* (I)], which reduce intracellular concentrations by expelling the antibiotic from the cell. This efflux-based strategy confers low-level resistance to 14- and 15-membered macrolides, and it is frequently observed in species such as *Streptococcus pneumoniae* and *Streptococcus pyogenes*. The second mechanism entails target site modification mediated by methylases encoded by *erm* family genes [including *erm*(A), *erm*(B), and *erm*(TR)]. These enzymes methylate the 23S rRNA of the bacterial ribosome, thereby inhibiting antibiotic binding. This modification confers high-level resistance to macrolides, lincosamides, and streptogramin B (the MLS_B_ phenotype), representing the most prevalent and clinically significant macrolide resistance determinants in streptococci and enterococci (Huang et al., 2016a).

The widespread dissemination of *erm* family genes in *S. agalactiae* and other streptococci is largely driven by integrative and conjugative elements (ICEs) (Morici et al., 2017; Chen et al., 2021; Yi et al., 2021). These mobile genetic elements excise from the chromosome, form a circular intermediate, and transfer between bacterial cells via conjugation machinery such as type IV secretion systems (T4SSs) and type IV coupling proteins (T4CPs) (Wozniak and Waldor, 2010). Following transfer, a site-specific integrase catalyses ICE integration into specific genomic sites, facilitating the efficient spread of antibiotic resistance genes (ARGs) (Partridge et al., 2018). Beyond antibiotic resistance, some ICEs can also transmit heavy metal resistance determinants, conferring related tolerance (Rodriguez-Blanco et al., 2012; Wang et al., 2025a).

Two groups of ICEs are currently recognized as common vehicles for the dissemination of *erm* genes in streptococci, namely the ICE*Sa*2603 and Tn*1806* families. The ICE*Sa*2603 family, first identified in *S. agalactiae* 2603V/R, is the most frequently reported group (Gawron-Burke and Clewell, 1982; Huang et al., 2016a; Yi et al., 2021). These elements often carry multiple antibiotic resistance genes and integrate site-specifically 3′ *rplL* gene, which encodes the 50S ribosomal subunit protein L7/L12. Representative members include ICE*Ssu*32457 from *Streptococcus suis*, carrying *aadE*-*aphA* (conferring aminoglycoside resistance), *erm*(B) and *tet*(40)-*tet*(O/W/32/O) (tetracycline resistance) (Palmieri et al., 2012), as well as ICE*Spn*529IQ from *S. pneumoniae*, which harbors *catQ* (chloramphenicol resistance), *tetM* (tetracycline resistance), *mef* (I), and *erm*(TR) (Mingoia et al., 2014). In addition, some ICE*Sa*2603-like family ICEs (characterized by distinct integrase modules and integration into alternative sites) also carry *erm* genes, such as ICE*Ssu*YZDH1 from *S. suis* (Yi et al., 2021) and ICE*Sag*37 from *S. agalactiae* (Zhou et al., 2017). The second group, Tn*1806*, was initially identified in *S. pneumoniae*, and it is characterized by its low-integration-site specificity and high transfer efficiency, promoting its dissemination across streptococci and enterococci (Camilli et al., 2008; Ambroset et al., 2016). However, ICEs are dynamic and modular. Genetic exchange and interaction can occur between different ICE groups, potentially giving rise to chimeric elements with novel characteristics and resistance profiles (Wang et al., 2025b). For instance, one ICE or mobile genetic element can integrate into a specific site within another, forming larger, more complex composite structures reminiscent of a “Russian doll” (Mingoia et al., 2016; Gawor et al., 2025). Although such composite elements/ICEs have rarely been documented, they can accumulate advantageous genes from multiple ICEs or other mobile genetic elements, thereby enhancing bacterial survival under diverse antibiotic pressures.

In this study, we analyzed the genetic context of antibiotic resistance genes in 16 macrolide-resistant isolates of *S. agalactiae* and identified a novel composite ICE. This ICE incorporates modules homologous to several previously characterized genetic elements, including an ICE*Sa*2603 family backbone, a Tn1806-like ICE, the *erm*(TR)-carrying element ICE*Sp*2907, and an uncharacterized *cadA*-carrying segment. These genetic elements are assembled at appropriate genomic locations according to their functional properties, resulting in a chimeric ICE that combines structural and functional features from distinct ancestral elements.

## 2 Materials and methods

### 2.1 Bacterial strains and susceptibility tests

This study included 47 non-duplicate clinical isolates of *S. agalactiae* collected and stored by the Shanghai Center for Clinical Laboratory (Shanghai, China) between 2021 and 2025. Among these, 16 isolates exhibited resistance to both erythromycin and clindamycin [minimum inhibitor concentration (MIC) > 1 mg/L]. Recipient strains for conjugation experiments were selected from the 47 *S. agalactiae* isolates. All bacterial strains were cultured in Todd–Hewitt (TH) broth at 37 °C for 8 h. Antimicrobial susceptibility testing was performed using the broth microdilution method, with breakpoints interpreted according to CLSI (the Clinical and Laboratory Standards Institute) guidelines. The reference strain *S. agalactiae* ATCC 13813 was obtained from the Huashan Hospital strain repository (Shanghai, China). The clinical isolates were partially collected through routine hospital laboratory procedures, and no additional personal information was involved in this study.

### 2.2 Genome comparative analysis and ICE identification

Genomic DNA was extracted from *S. agalactiae* using the QIAGEN Midi Kit (Qiagen, Hilden, Germany) and sequenced by next-generation sequencing (NGS) at BGI Genomics (Shenzhen, China) using HiSeq X (Illumina, San Diego, CA, USA). High-quality reads were assembled with SPAdes (Bankevich et al., 2012), and the assembled sequences were annotated through the RAST online server (rast.nmpdr.org) (Aziz et al., 2008). Contigs were ordered against the complete reference genome *S. agalactiae* 2603V/R using the “Order Contigs” function in Mauve v2.4.0 (Gawron-Burke and Clewell, 1982; Darling et al., 2004). The reordered genomes were then aligned and visualized against the reference using the “Comparison” function in Mauve, enabling preliminary localization of large genetic elements. Putative mobile genetic elements were identified by screening for genes encoding T4SSs, T4CPs, relaxase, and integrase. These candidate regions were further analyzed using ICEfinder to confirm their classification as ICEs.

### 2.3 Amplification experiments

PCR assays were performed for three objectives (Supplementary Table 1): to screen candidate recipient strains by detecting the presence of an unoccupied *rplL* gene; to amplify conserved ICE genes in trans-conjugants for preliminary confirmation of ICE integration; and to examine ICE integration, excision, and circularization using the primer pairs P1/P2 and P3/P4 to detect integration, the primer pair P1/P4 to detect excision, and the inverse primer pair P2/P3 to detect circular intermediates.

### 2.4 Conjugative transfer assays

Recipient strains were selected from clinical isolates based on two criteria: a resistance profile distinct from that of the donor strain and the presence of at least one unoccupied ICE integration site. These characteristics were initially assessed using antimicrobial susceptibility testing and PCR and subsequently confirmed by whole-genome sequencing.

Conjugation was performed using a filter mating method as previously described (Palmieri et al., 2012). Fifty mL Donor and 250 mL recipient strains were grown to the late-log phase, normalized to the same CFU, and mixed at a donor-to-recipient ratio of 1:5. Prior to mixing, 200 μL of the donor culture was sampled for CFU enumeration to calculate the conjugation frequency. A nitrocellulose membrane was cut to an appropriate size, treated with 10 μg/mL DNase to prevent transformation-mediated DNA transfer, and placed on a TH agar plate. The mixed culture was pelleted by centrifugation, resuspended, and evenly spread onto the DNase-treated membrane, followed by incubation at 37 °C for 4 h. After incubation, cells were harvested from the membrane, serially diluted, and plated onto TH agar containing 50 μg/mL erythromycin, 50 μg/mL clindamycin, and 20 μg/mL levofloxacin. Transconjugant colonies were counted after 2–3 days of incubation and randomly selected for validation.

The conjugation frequency was calculated as the number of transconjugants divided by the number of donor cells. Donor count: 100 μL culture was taken from a 50 mL donor culture and serially diluted 10-fold in 1 × PBS for 6-10 times. After thorough mixing, 100 μL dilution was spread evenly onto counting plates. Colony counts between 30 and 300 were considered valid. Transconjugant count: After mating, cells were collected from the nitrocellulose membrane using a sterile swab and resuspended. The suspension was then diluted 10-fold, 100-fold, or used undiluted, and plated onto transconjugant selective medium. Plates with 30-300 colonies were considered valid. If the undiluted suspension yielded transconjugants but fewer than 30 colonies, the transfer was considered successful, but the frequency was too low to be calculated accurately.

### 2.5 Growth curve analysis under cadmium stress

To evaluate the growth dynamics of the recipient and transconjugants under cadmium stress, growth curves were generated in TH broth with or without cadmium as previous described with minor modification (Parsons et al., 2017). The recipient and transconjugant strains were inoculated into two culture conditions: unsupplemented TH broth and TH broth supplemented with 15 μM CdCl2 to induce metal stress. Cultures were incubated at 37 °C with shaking at 160 rpm, and optical density at 600 nm was measured hourly using a microplate reader until the stationary phase was reached. Growth curves were plotted using GraphPad Prism (GraphPad, Boston, MA, USA), and the growth of transconjugants was compared with that of the recipient strain under cadmium stress. All experiments were performed in at least two independent biological replicates to ensure reproducibility.

### 2.6 Bioinformatics identification of ICEs similar to composite ICE*Sag*39

The core genes of ICE*Sag*39, including the tandem serine integrase, relaxase, and conjugation modules (including T4SS and T4CP cluster) of the Tn*1806* family, along with the tyrosine integrase, relaxase, and conjugation modules of the ICE*Sa*2603 family, were used as templates for BlastN analysis in Nucleotide Collection and Whole-genome shotgun contigs databases from NCBI. According to the criteria defined by ICEFinder (db-mml.sjtu.edu.cn/ICEberg) and previous reports (Ambroset et al., 2016; Huang et al., 2019), a genomic region was considered to harbor an ICE similar to ICE*Sag*39 if it contained genes homologous to these core genes (>60% nucleotide identity); the maximum distance between these genes was < 100 kb; Minimum sequence coverage of BLAST hits >25%. In contigs database, a sequenced strain was considered to contain the composite ICE only when the complete element was identified on one scaffold/contig. Boundaries and attachment sites of ICEs were manually checked by terminal integrase and *rplL* genes. ICE-carried AMR genes were identified as previously described.

### 2.7 GeneBank accession number

Sequence data generated in this study have been deposited in the NCBI GenBank, including the NGS drafts of 16 erythromycin- and clindamycin-resistant *S. agalactiae* isolates, the complete genomes of recipient *S. agalactiae Sag*R272 and *Sag*R31, and the NGS draft of transconjugants *Sag*R272_TC and *Sag*R31_TC. Public data used in this study included Tn*1806*, ICE*Sp*2907, ICE*Spy*009, ICE*Spy*009, and the complete genome of *S. agalactiae* 2603V/R.

## 3 Results

### 3.1 Characterization of erythromycin- and clindamycin-resistant isolates

The NGS data of 16 erythromycin- and clindamycin-resistant *S. agalactiae* isolates revealed diverse phenotypic and genotypic profiles (Table 1). Ten carried the *erm*(B) gene, and one contained the *erm*(TR) gene. Three isolates harbored the macrolide resistance genes *mef* (E) and/or mel, whereas tetracycline resistance, associated with the *tet*(M) gene, was observed in seven isolates.

**Table 1:** Phenotypic, genotypic, and other characteristics of the 16 erythromycin- and clindamycin-resistant *Streptococcus agalactiae* isolates.

**Strains**	**Year**	**MIC (mg/L)**					**Resistance genotype**	**ARGs-carrying genetic elements**	**Serotype**	**Accession number**
		**ERY**	**CLI**	**TET**	**LEV**	**CHL**				
*Sag*191	2021	>256	>256	128	≤0.5	≤0.5	*erm*(B), *tetM*	Tn*1806-*like ICE	ST1	-
*Sag*199	2021	>256	>256	≤0.5	≤0.5	≤0.5	*erm*(B)	Tn*1806-*like ICE	ST10	-
*Sag*228	2022	>256	>256	≤0.5	≤0.5	≤0.5	*erm*(B)	Tn*1806-*like ICE	ST10	-
*Sag*257	2022	64	>256	64	32	1	*mef(E)-mel, tetM*	ICE*Sa*2603, Tn*916*	ST19	JBAPEL000000000
*Sag*273	2022	>256	>256	≤0.5	≤0.5	≤0.5	*erm*(B), *mel*	Tn*1806-*like ICE	ST10	-
*Sag*2111	2022	4	2	32	≤0.5	≤0.5	*tetM*	Tn*916*	ST23	JBAPEO000000000
*Sag*2142	2022	>256	>256	≤0.5	≤0.5	≤0.5	*erm*(B)	Tn*1806-*like ICE	ST10	-
*Sag*2157	2022	>256	>256	≤0.5	≤0.5	≤0.5	*erm*(B)	Tn*1806-*like ICE	ST10	-
*Sag*2167	2022	>256	>256	≤0.5	≤0.5	≤0.5	*erm*(B)	Tn*1806-*like ICE	ST10	JBAPEQ000000000
*Sag*35	2023	>256	>256	≤0.5	≤0.5	≤0.5	*erm*(B)	Tn*1806-*like ICE	ST10	-
***Sag*** **39**	**2023**	**>256**	**>256**	**>256**	**≤0.5**	**≤0.5**	***erm*****(TR)**, ***tetM***	**ICE** ***Sag*** **39, Tn** ***916***	**ST19**	**JBAPEP000000000**
*Sag*3155	2023	>256	>256	1	≤0.5	≤0.5	*erm*(B)	Tn*916*	ST10	-
*Sag*3220	2023	>256	256	>256	≤0.5	≤0.5	*mef(E)-mel, tetM*	ICE*Sa*2603	ST17	JBAPER000000000
*Sag*4240	2024	>256	>256	≤0.5	2	≤0.5	*erm*(B)	Tn*1806-*like ICE	ST10	JBAPES000000000
*Sag*4272	2024	1	1	>256	≤0.5	≤0.5	*tetM*	Tn*916*	ST10	JBAPEB000000000
*Sag*516	2025	1	2	32	1	1	*tetM*	Tn*916*	ST10	JBAPEH000000000

MIC, minimum inhibitory concentration; ERY, erythromycin; CLI, clindamycin; TET, tetracycline; LEV, levofloxacin; CHL, chloramphenicol; ARG, antibiotic resistance gene. The “-” indicate that the corresponding sequence was not essential for this study and therefore has not been uploaded to the NCBI database. Bolded entries represent the primary subjects, host of ICESag39, of this investigation.

Multi-locus sequence typing identified ST10 as the most predominant sequence type (11 isolates), followed by ST19 (two isolates). ST1, ST17, and ST23 (one isolate each) were also detected.

### 3.2 Identification of antibiotic resistance gene-carrying genetic elements

**Figure 1:**
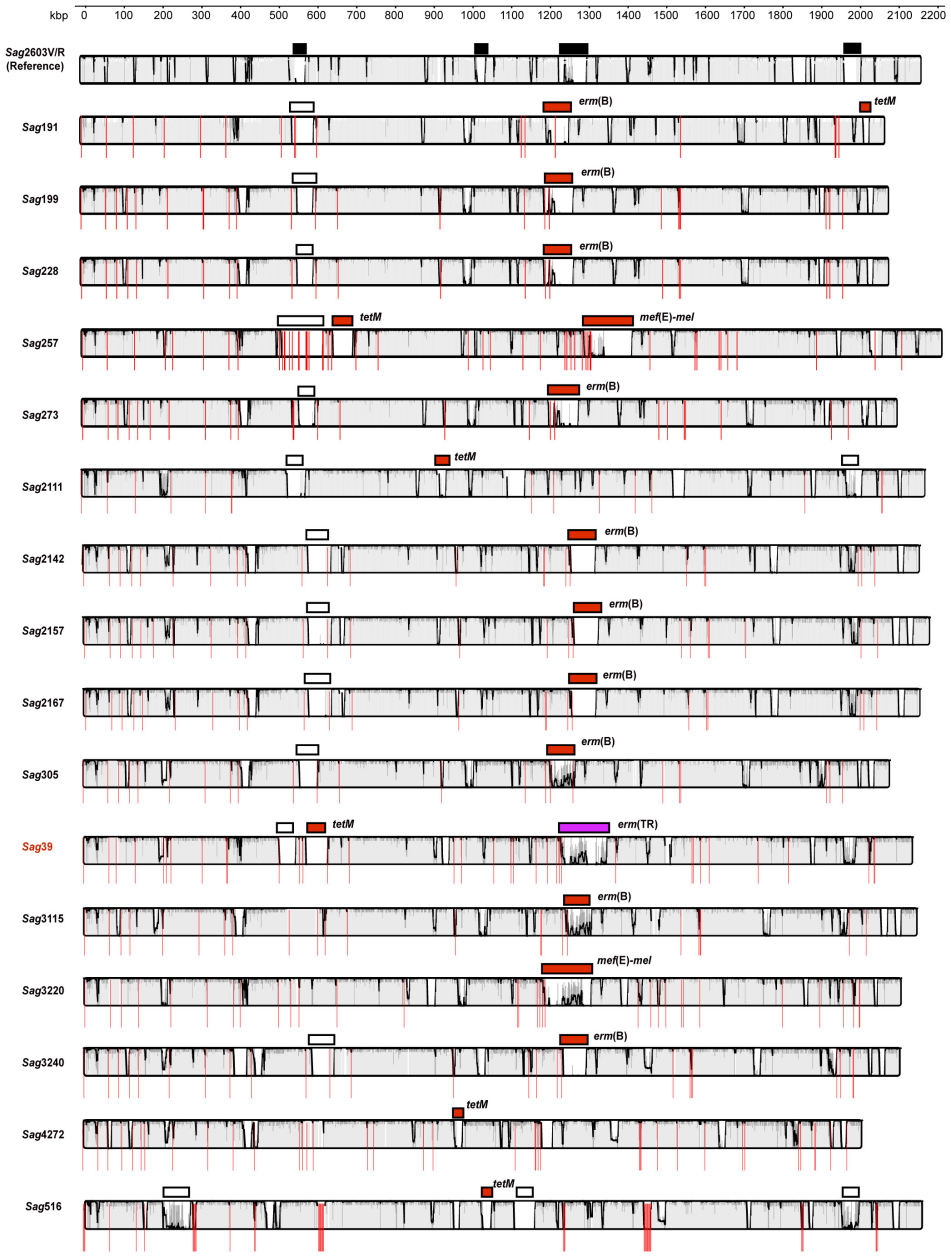
Linear comparison of 16 whole-genome shotgun assemblies of *Streptococcus agalactiae* against the reference strain 2603V/R (AE009948). Conserved syntenic regions are presented as solid colored lines. Discontinuities likely correspond to insertions of exogenous mobile genetic elements (MGEs). Resistance gene-carrying elements are indicated by red rectangles. Other MGEs (lacking known resistance genes or uncharacterized) are presented in white. ICE*Sag*39 is highlighted in purple, and its host genome is marked in red. The reference genome includes four well-characterized MGEs denoted by black rectangles (left to right): lambdaSa1 (558,765–559,346 bp), Tn916 (923,439–941,360 bp), ICESa2603 (1,256,480–1,311,228 bp), and lambdaSa2 (1,833,201–1,867,137 bp). Red vertical lines separate individual sequencing scaffolds.

To localize antibiotic resistance genes and identify their associated genetic elements, we compared the genomes of the 16 *S. agalactiae* isolates with that of the reference strain 2603V/R (Huang et al., 2016b). This analysis identified 35 major genomic regions of difference, 19 of which contained antibiotic resistance genes along with integrase genes, suggesting integration of mobile genetic elements into the genome (Figure 1). Comparative analysis using GenBank classified the antibiotic resistance gene-carrying elements into several known families: the Tn*1806* family carrying *erm*(B), the Tn*916* family carrying *tet*(M), and the ICE*Sa*2603 family carrying *mef* (E)-*mel* (Table 1). A novel composite genetic structure carrying *erm*(TR) was identified in *S. agalactiae Sag*39, and it was formed by the integration of Tn*1806*- and ICE*Sa*2603-like ICE 3′ *rplL* gene. This element also carried the cadmium resistance gene *cadA*, and because of the presence of multiple integrase and conjugation-related genes, it was classified as a composite ICE and designated ICE*Sag*39.

### 3.3 Characterization of ICE*Sag*39 and the internal Tn*1806*-like ICE

**Figure 2:**
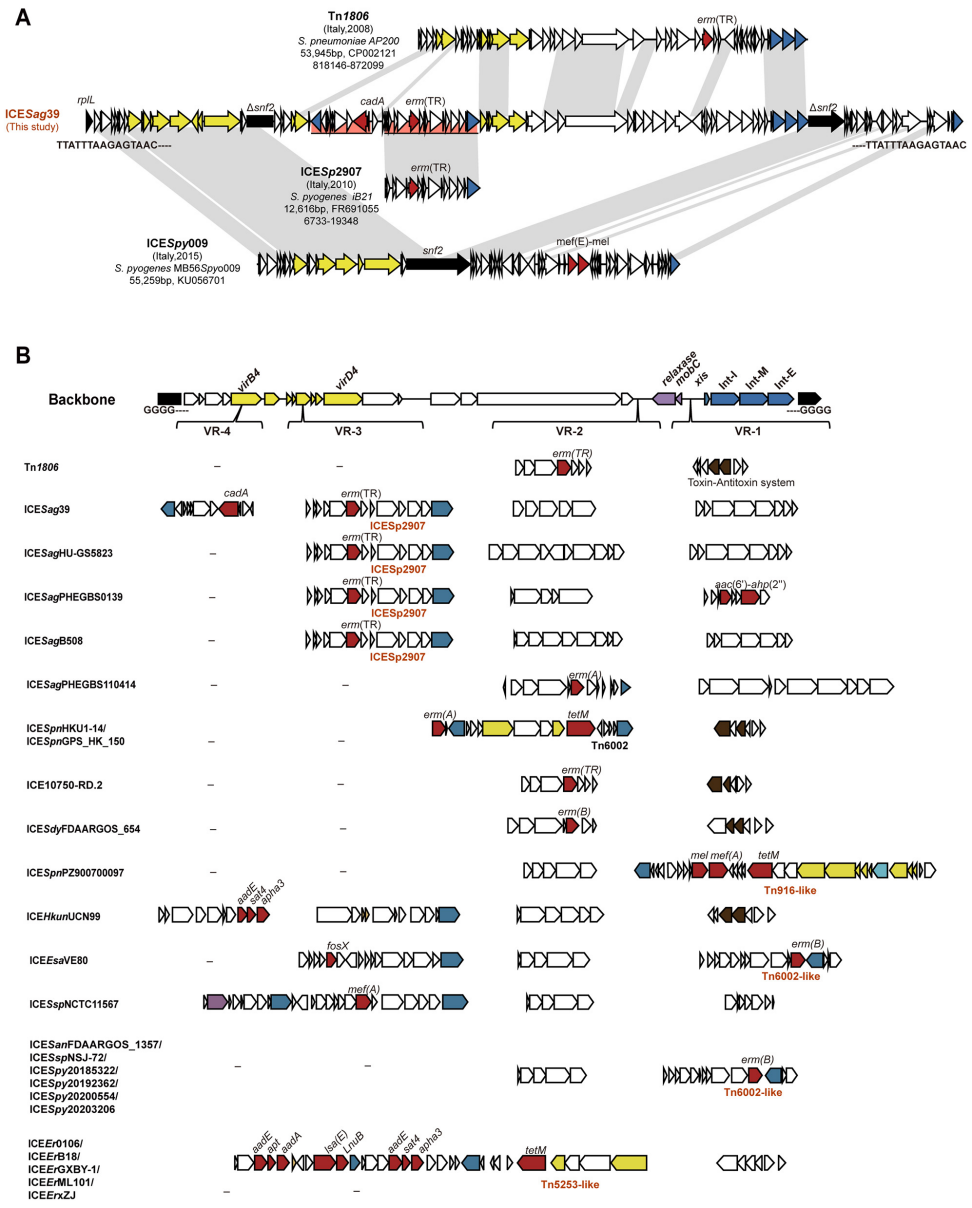
Genetic context of ICE*Sag*39. **(A)** Comparative analysis of ICE*Sag*39 with known integrative and conjugative elements (ICEs) from the NCBI database, including Tn*1806* (CP002121, 818,146–872,099 bp), ICE*Sp*2907 (FR691055, 6733–19,348 bp), and ICE*Spy*009 (KU056701). The direct repeat sequences flanking the ICE are TTATTTAAGAGTAAC–TTATTTAAGAGTAAC. Additional regions are highlighted in different background colors. (**B)** Structure of internal Tn*1806*-family ICEs displaying conserved modules and variable regions (VR1–VR4) based on an alignment of 25 antibiotic resistance gene-carrying ICEs. Open-reading frames are colored by functional category: integrase/transposase/recombinase (dark blue), relaxase and MobC (purple), type IV secretion system-related genes (yellow), resistance genes (red), host gene interrupted by ICE insertion (black), and toxin-antitoxin systems (brown). Truncated genes are indicated by triangles. Light gray shading denotes regions sharing >60% sequence similarity.

ICE*Sag*39 measured 112,852 bp in length and featured 102 open-reading frames and 35% GC content. The entire ICE was flanked by direct repeats (TTATTTAAGAGTAAC–TTATTTAAGAGTAAC) 3′ *rplL* gene. Comparative genomic analysis revealed that ICE*Sag*39 comprises four modules (Figure 2A). The first module shared high similarity with ICE*Spy*009, a member of the ICE*Sa*2603 family, but lacked the *mef* (E)-*mel* gene cluster present in *S. pyogenes* (Del Grosso et al., 2016). A Tn*1806*-like ICE inserted within the *snf2* (methyltransferase encoding the sucrose non-fermenting 2 protein) gene of the ICE*Spy*009 backbone constituted the second module. Notably, this internal Tn*1806*-like ICE itself carried two additional mobile genetic units: ICE*Sp*2907, which lacks a conjugation system but carries *erm*(TR) (Giovanetti et al., 2012), and an uncharacterized element harboring *cadA*.

To explore the structural organization and integration hotspots for exogenous DNA, we analyzed the relatively variable Tn*1806*-like ICE. This analysis involved 41 additional ICEs with high sequence similarity to Tn*1806* from NCBI databases, and 25 of these ICEs carried at least one antibiotic resistance genes (Supplementary Table 2). These elements integrate with low specificity into a 4-bp direct repeat sequence (GGGG) within *snf2* or diverse methyltransferase encoding genes, truncating the target gene upon insertion. Beyond the conserved integrase and conjugation modules, four variable regions (VR1–VR4) were identified as hotspots for the integration of foreign genetic elements (Figure 2B, Supplementary Table 3). VR1 and VR2 frequently harbored *erm*(A) and *erm*(B), along with the aminoglycoside resistance gene *aac*(6′)-*aph*(2″) in some cases. Entire mobile elements such as Tn*5253* (Mingoia et al., 2014), Tn*916* (Rice, 1998), and Tn*6002* (Brenciani et al., 2007) were also identified within these regions. VR3 served as a specific integration site for ICE*Sp*2907, which carries *erm*(TR). VR4, uniquely identified in ICE*Sag*39, contained the *cadA*-carrying genetic element.

### 3.4 Excision and circulation of ICE*Sag*39 and the internal Tn*1806*-like ICE

**Figure 3:**
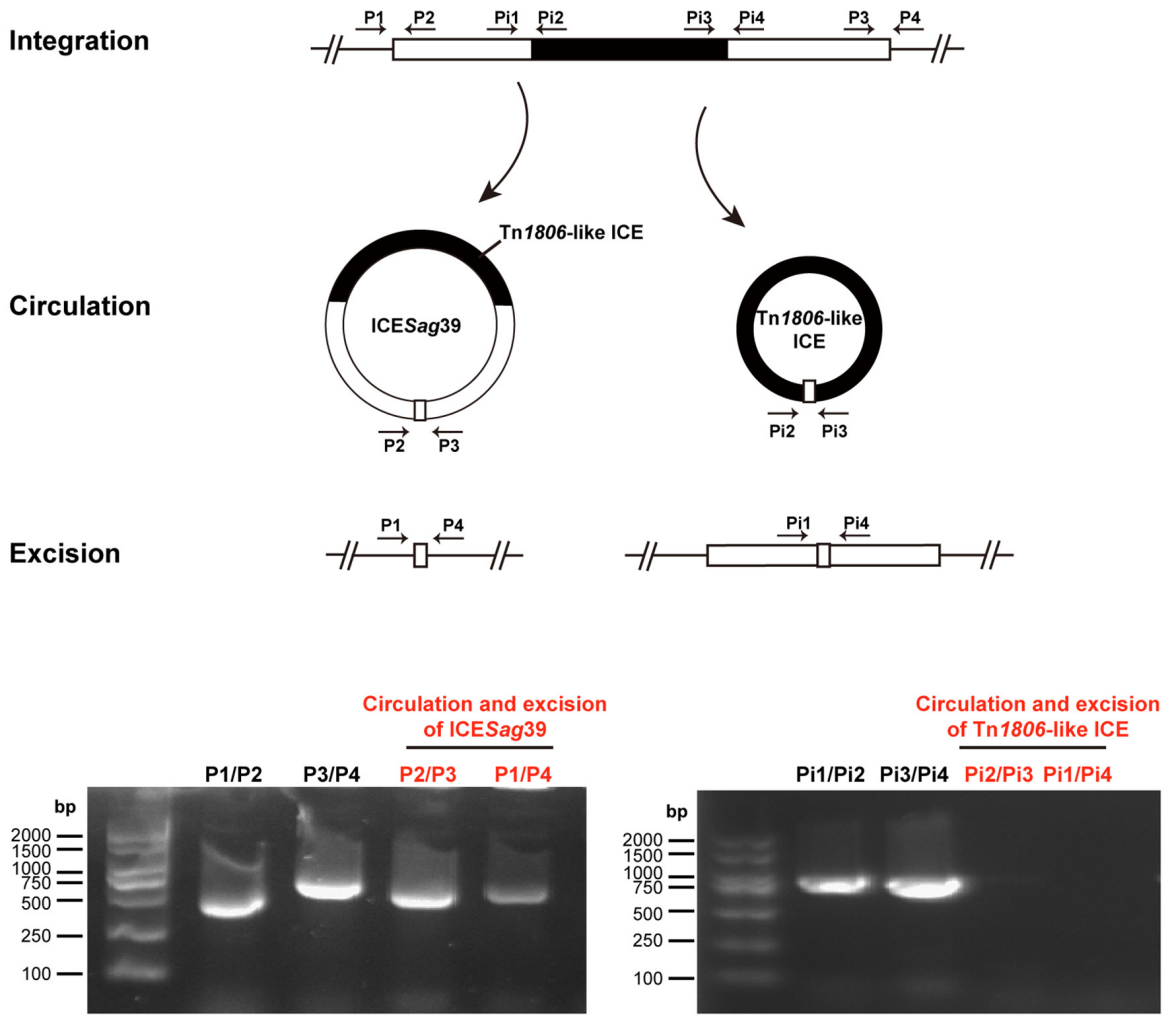
Diagram and PCR detection of integration and excision/circularization of ICE*Sag*39 and an internal Tn*1806*-like integrative and conjugative element (ICE). Black rectangles represent the Tn*1806*-like ICE within ICE*Sag*39, and the combined white and black rectangles represent the composite ICE*Sag*39. The primers used are indicated by black arrows. The bottom panel presents the PCR results, and the PCR marker is Vazyme DL2000 DNA. The primer pairs P2/P3 and P1/P4 represent circulation and excision, the primer pairs P1/P2 and P3/P4 represent integration of ICE*Sag*39, the primer pairs Pi2/Pi3 and Pi1/Pi4 represent circulation and excision, and the primer pairs Pi1/Pi2 and Pi3/Pi4 represent integration of the internal Tn*1806*-like ICE.

Chromosomal excision and subsequent circularization are essential for the rolling circle replication and conjugative transfer of ICEs. To preliminarily assess the transfer potential of ICE*Sag*39 and the embedded Tn*1806*-like ICE, we designed PCR primers to detect these processes. Amplification with the primer pairs P1/P2 and P3/P4 as well as Pi1/Pi2 and Pi3/Pi4 (targeting ICE-flanking regions) yielded positive products for both ICE*Sag*39 and the internal Tn*1806*-like ICE (Figure 3), confirming their chromosomal integration. However, only ICE*Sag*39 produced faint PCR products with the primer pairs P1/P4 (excision) and P2/P3 (circular intermediate), whereas corresponding amplicons Pi1/Pi4 and Pi2/Pi3 were undetectable for the Tn*1806*-like ICE (Figure 3). These results suggest that ICE*Sag*39 is capable of excising and forming circular intermediates, whereas the internal Tn*1806*-like ICE appears incapable of autonomous excision, or its transfer frequency is too low to be detected by PCR.

### 3.5 Transferability of ICE*Sag*39

**Figure 4:**
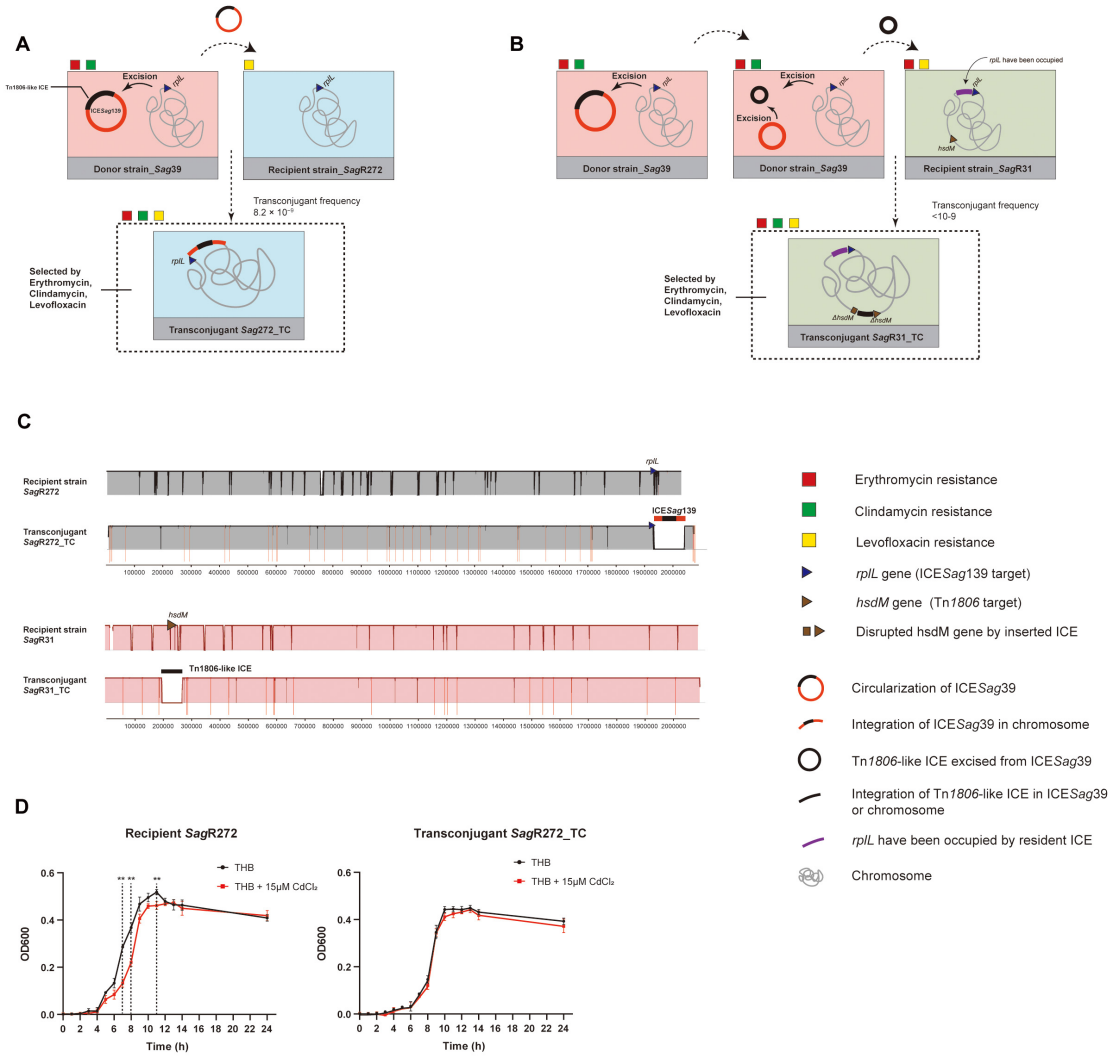
Conjugation transfer experiments of ICE*Sag*39. **(A, B)**, Schematics of the experimental workflow for conjugative transfer of mobile genetic elements from donor *S. agalactiae Sag*39 to recipient strains *Sag*R272 and *Sag*R31, respectively. **(C)** Linear comparison of the recipient and transconjugant genomes confirming the specific integration of the target integrative conjugative element (ICE) into the recipient's genome. **(D)** Impact of ICE*Sag*139 (carrying *cadA*) on bacterial growth under cadmium stress. Growth curves of the recipient strain and transconjugant in Todd–Hewitt broth with or without 15 μM CdCl2 monitored over 24 h. The recipient strain displayed significant growth impairment in the presence of CdCl2, with notable differences observed at 7, 8, and 11 h (Repeated Measures ANOVA, ^**^p < 0.001). The growth of the transconjugant remained unaffected by cadmium.

*S. agalactiae Sag*39 carrying ICE*Sag*39 was used as a donor (resistant to erythromycin, clindamycin, but sensitive to levofloxacin). The recipient strain, *S. agalactiae Sag*R272 (sensitive to erythromycin, clindamycin, but resistant to levofloxacin), which possesses an unoccupied *rplL* gene for ICE*Sag*39, was selected (Figure 4A). In mating experiments, the complete ICE*Sag*39 was transferred from the donor to the recipient at a frequency of 8.2 × 10^−9^ (Table 2). Three randomly selected transconjugants acquired high-level resistance to erythromycin and clindamycin while retaining the recipient's resistance to levofloxacin. Under cadmium stress (15 μM CdCl2 in TH broth), transconjugants exhibited significantly better growth than the recipient (Figure 4D), suggesting that transfer of cadmium resistance. Whole-genome sequencing confirmed integration of ICE*Sag*39 downstream of the *rplL* gene in the transconjugants *Sag*R272_TC comparing with recipient *Sag*R272, as expected (Figure 4C).

**Table 2:** Characteristics of the donor, recipient, and related transconjugants.

**Strains**	**Purpose**	**MIC (**μ**g/mL)**				**Transfer frequency**	**Putative integration sites**	**Transfer ICE**	**Accession No**.	**Objective ICE location**
		**ERY**	**CLI**	**TET**	**LEV**					
*Sag39*	Donor	**>256**	**256**	32	≤0.5				JBAPEP000000000	Sequence02, 12144-62118
*Sag*R272	Recipient	≤0.5	1	128	**32**	8.2 × 10^−9^	*rplL*	ICE*Sag*39	CM130719	
*Sag*R272_TC	Transconjugant	**>256**	**>256**	128	**32**				JBRZQM000000000	
*Sag*R31	Recipient	4	≤0.5	4	**64**	<10^−9^	*hsdM*	Tn*1806*-like ICE	CP138369	
*Sag*R31_TC	Transconjugant	>256	**256**	4	**64**				JBRZQN000000000	

The bold values were obtained by antibiotic resistance screening via a conjugation transfer experiment. MIC, minimum inhibitory concentration; ERY, erythromycin; CLI, clindamycin; TET, tetracycline; LEV, levofloxacin; ICE, integrative and conjugative element.

To assess whether the internal Tn*1806*-like ICE could excise and transfer independently, another isolate, *S. agalactiae Sag*R31 (sensitive to clindamycin, but resistant to erythromycin and levofloxacin), was used as the recipient. This strain contains an *hsdM* gene (encoding type I methyltransferase), a known integration site for Tn*1806* family ICEs, and the *rplL* site has been occupied by a resident ICE (Figure 4B). After three independent mating experiments, the Tn*1806*-like ICE transferred only once. The transconjugant counts obtained were 0, 0, and 7, respectively, none of which reached the minimum countable threshold of 30 and were therefore considered as < 30; the donor counts in the mating experiments were 3.4 × 10^10^ CFU, 4.1 × 10^10^ CFU, and 3.2 × 10^10^ CFU. Therefore, calculated conjugation transfer frequency was < 10^−9^. Whole-genome sequencing confirmed Tn*1806*-like ICE were compensatorily integrated into *hsdM* genes in recipient *Sag*R31 (Figure 4C).

### 3.6 Prevalence of composite genomic elements resembling ICE*Sag*39

We screened the NCBI GenBank database using BlastN to identify composite ICEs structurally similar to ICE*Sag*39 in *S. agalactiae*. Details of the identified ICE, including the host strain, accession number, genomic location, length, and carried antibiotic resistance genes, are summarized in Supplementary Table 4. In total, 199 ICE*Sag*39-like ICEs were identified, including seven from complete genomes and 192 from whole-genome shotgun contigs, all integrated 3′ *rplL* gene. These strains were unloaded from different years (2012–2025), with 74.4% (148/199) from the United States, 16.6% (33/199) from China, 4.0% (8/199) from South Korea, 2.0% (4/199) from Brazil, 1.5% (3/199) from the United Kingdom, 1.0% (2/199) from Italy, and 0.5% (1/199) from Japan. Their sizes ranged from 90,473 to 138,541 bp. Analysis of resistance gene carriage revealed that 62.8% (125/199) of these ICEs harbored both *erm*(TR) and *cadA*, 22.1% (44/199) carried only c*adA*, and 4.5% (9/199) carried only *erm*(TR). Additionally, 1.5% (3/199) carried *aac*(6′)-*aph*(2″) together with *erm*(TR), whereas 7.0% (14/199) possessed *erm*(TR) along with *catQ*–*mef* (I). Four ICEs lacked any known resistance genes. Notably, nearly all resistance genes were located within the embedded Tn*1806*-like ICE module of these composite elements.

## 4 Discussion

Macrolide resistance determinants, such as erm family genes and the heavy metal resistance gene *cadA*, are widely recognized as common resistance factors. The *erm*(B) gene in particular exhibits a detection rate as high as 89.3% in *S. agalactiae*, posing a challenge of global significance (Moroi et al., 2019; Hatrongjit et al., 2025). In 2019, the US Centers for Disease Control and Prevention identified 21 antibiotic resistance threats, classifying clindamycin-resistant *S. agalactiae* and erythromycin-resistant *S. pyogenes* at the “concerning” level (CDC, 2019). Moreover, *cadA* has frequently been detected on plasmids from *erm*(B)- or *mef* (A)-positive isolates, indicating a potential linkage between these commonly co-occurring resistance genes (Costa et al., 2016).

This study identified a novel composite ICE characterized by a nested architecture, formed by an ICE*Sa*2603 family backbone integrated with a Tn*1806*-like ICE, spanning 112,852 bp in length. The embedded Tn*1806*-like ICE constitutes an autonomous genetic module featuring four variable regions (VR1–VR4) that act as insertion hotspots for additional resistance determinants. Specifically, ICE*Sp*2907 carrying *erm*(TR) and an uncharacterized *cadA*-carrying element were identified within VR3 and VR4, respectively (Giovanetti et al., 2012). Comparative genomic analysis further revealed that VR1 and VR2 might harbor resistance genes such as *aac*(6′)-*aph*(2″) (aminoglycoside), *erm*(B) (MLS_B_), and *tet*(M) and potentially accommodate entire mobile elements such as Tn*6002*, Tn*916*, and Tn*5253*. The backbone of ICE*Sag*39 represents a typical member of the ICE*Sa*2603 family, as evidenced by its tyrosine integrase, specific integration site *rplL* gene, and flanking 15-bp direct repeat sequences (Marini et al., 2015; Huang et al., 2016a). This backbone provides a stable genomic platform for the acquisition of exogenous DNA and rplL as the defined integration locus. By contrast, the embedded Tn*1806*-like ICE substantially expands the accessory gene content available to the ICE*Sa*2603 family by serving as an insertion site for smaller genetic elements. This Russian doll-like nesting of mobile genetic elements produced the formation of the composite ICE ICE*Sag*39, facilitating the co-localization and potential co-transfer of diverse resistance genes.

Through a systematic comparative analysis of Tn*1806*-like ICEs, this study first expanded the recognized diversity of this ICE group and elucidated the molecular basis for the formation of the composite ICE ICE*Sag*39. The Tn*1806*-like ICE identified in this study was integrated within the 6-kb (GGG–GGG) *snf2* gene of ICE*Sag*39, an integration site that differs from the previously characterized hsdM (encoding a type I methyltransferase) target observed in Tn*1806* and ICE*Spy*10750 (Beres and Musser, 2007; Camilli et al., 2008; Ambroset et al., 2016). An expanded screening of Tn*1806*-like ICEs identified four additional Tn*1806-*like ICEs from Gen Bank also integrated within the *snf2* gene, indicating that *snf2* represents a previously overlooked integration site for this ICE family. Notably, *snf2* has been reported as a conserved gene in the ICE*Sa*2603 family, and it is also present in enterococcal pheromone-responsive plasmids such as pBET_5 (Shan et al., 2022; Yang et al., 2022). The targeting of *snf2* as an integration site strongly suggests that the composite structure of ICE*Sag*39, arising from the combination of ICE*Sa*2603 and Tn*1806*-like ICEs, is not an occasional event, but reflects a high-affinity interaction. This interpretation is further supported by the identification of 199 similar ICE*Sag*39-like ICEs in public databases. However, no composite plasmid-ICE structures associated with Tn*1806*-like ICEs or enterococcal pheromone-responsive plasmids were detected, despite the presence of the *snf2* gene in such plasmids and prior evidence supporting the interspecies transferability of Tn*1806*-like ICEs. This absence might reflect specific genetic or physiological barriers that limit the formation of such composite, a possibility that remains to be investigated.

ICE*Sag*39 represents a novel class of composite ICEs that was demonstrated for the first time in this study to possess a modular architecture and to be transferable to other *Sagalactiae* strains. The embedded Tn*1806*-like ICE, which acts as a primary carrier of antibiotic and heavy metal resistance genes, is not a fixed component of the structure. Conjugation experiments confirmed that individual internal ICEs within ICE*Sag*39 can be excised from the composite and transferred independently. However, detection of circular intermediates and conjugation frequency indicated that the transfer efficiency of internal ICEs is remarkably low, suggesting that ICE*Sag*39 generally maintains its structural integrity under most conditions. In situations unfavorable for the transfer of the composite ICE, such as when its length imposes a high fitness cost or when suitable integration sites are absent in the recipient, the ability of internal Tn*1806*-like ICEs to transfer independently might provide an alternative dissemination pathway. This structural flexibility highlights a distinct adaptive advantage of composite ICEs over simple genetic elements. The assembly of *erm*(TR) and *cadA* within a single transferable ICE provides important insights into the evolution of ICE diversity and the horizontal dissemination of resistance determinants. Integrating these advantageous genes into a large, mobile composite ICE both increases the risk of multidrug-resistant “superbug” emergence and accelerates the diversification of bacterial genomes.

## Data Availability

The datasets presented in this study can be found in online repositories. The names of the repository/repositories and accession number(s) can be found in the article/Supplementary material.
